# Effectiveness of the Horn of Africa Polio Outbreak Coordination Office in Nairobi, Kenya

**DOI:** 10.29245/2578-3009/2021/S2.1114

**Published:** 2021-04-15

**Authors:** Samuel Okiror, Hemant Shukla, Bob Davis, Brigitte Toure, Rustum Hydarov, John Burton, Subroto Mukherjee, Bal Ram Bhui, Mercy Lutukai, Chidiadi Nwogu, Joseph Okeibunor

**Affiliations:** 1 WHO Horn of Africa Coordination Office (HOA), Nairobi KENYA; 2 WHO Headquarters, Geneva; 3 American Red Cross, Nairobi Kenya; 4 UNICEF, Nairobi Kenya; 5 UNHCR, Nairobi Kenya; 6 USAID Technical Officer; 7 WHO Regional Office for Africa, Brazzaville, Congo; 8 CORE Group Regional Office Nairobi

**Keywords:** Coordination, Horn of Africa, Polio outbreaks, Response

## Abstract

**Background:**

The WPV1, first detected in Somalia in April 2013, quickly spread to Kenya and Ethiopia and triggered a multi-country coordinated effort. In February 2014, a formal HoA Polio Outbreak Coordination Office was established by WHO AFRO and WHO EMRO in Nairobi to provide technical and managerial leadership. An independent assessment was conducted to ascertain the usefulness of the HoA Coordination in response to the outbreaks.

**Methods:**

The independent assessment team conducted desk review of the rules and guidelines forming the HoA Coordination office and committee. It also reviewed minutes of meetings and interviewed various stakeholders at the Regional levels.

**Results:**

This independent review of the work of the office, in September 2016, showed that the office was fully functional and had benefited from financial and technical support from regional and global GPEI partners. The office is based in the WHO Kenya Country Office which also provides administrative, logistics and until August 2016, data management support. The close working relationship with technical partners ensured alignment and close coordination of outbreak response activities. The mechanism also allowed partners to identify areas of work based on their expertise and avoided duplication of efforts at the local level. Overall, the office was effective in close monitoring of implementation of the outbreak response, strengthening of cross-border activities, monitoring implementation of the TAG recommendations, improving SIA planning and quality, and expanding independent monitoring in Somalia and South Sudan. Key constraints included limited office space for day-to-day operations, and disruption of some activities due to interruption of contracts of technical staff. However, the closure of the HoA outbreak in August 2015 led to some complacency, resulting in a lost sense of urgency, negatively impacting the coordination.

**Conclusions:**

The HoA Coordination Office should continue to function into the foreseeable future. To ensure sustainability of activities, the technical staff should be given contracts for a minimum of 12 months. The Office should reintroduce and schedule the Joint Polio Outbreak Response team meetings at least once every three months.

## Introduction

The Global Polio Eradication Initiative (GPEI) was set up with the primary goal of completing the eradication and containment of all polioviruses, such that no child ever again suffers paralytic poliomyelitis^1^. Following the adoption of the mandate of the GPEI, by the World Health Assembly, the World Health Organization (WHO) and its partners made good efforts to accelerate the interruption of polio viruses globally^2–6^. However, between 2013 and 2014, the Horn of Africa (HOA) experienced the most devastating outbreaks of wild poliovirus typel (WPV1), which started in April 2013, with detection of a case in Banadir region of Somalia and spread to other districts in the country as well as neighboring countries^7–9^.

The outbreak response called for a massive multicountry coordinated effort. The need for strong coordination became clearer when virus spread from Somalia across the region to Kenya and Ethiopia. In August 2013, with the support of the Bill and Melinda Gates Foundation (BMGF) and CDC-Atlanta, a HOA Polio Outbreak coordinator was placed in the UNICEF Eastern and Southern Africa Regional Office (ESARO), to initiate and support inter-regional and country interactions. The ten affected countries established a platform to make collective decisions; to synchronize activities and organize cross border collaboration for polio campaigns; and to develop a joint WHO/UNICEF HOA donor appeal in the fall of 2013.

In October 2013, at its 8^th^ meeting, the Independent Monitoring Board (IMB) for the Polio Eradication Initiative reviewed the status of the HOA outbreak response. The IMB went on to state that: “Polio in the Horn of Africa needs to be treated as a public health emergency, with commensurate high-level political commitment, unambiguous and coordinated program leadership, plentiful support to the affected countries, and thoroughness of action... the organizational structure of partner agencies has impeded a coordinated approach”. As a result, coordination was further strengthened by the creation of a formal structure, called the Horn of Africa Polio Outbreak Coordination Office based at WHO Kenya. In February 2014, a Coordinator was duly appointed by both WHO/AFRO and WHO/EMRO, and relocated to Nairobi, Kenya. The office functioned until end of 2016 when the Global Polio Eradication Initiative (GPEI) was sure that the outbreak of wild poliovirus that the office was created to interrupt was over.

The Office was established on a *“temporary basis”* to provide technical and managerial leadership of a joint HOA Polio Outbreak Response Team that comprised technical officers from GPEI Partner agencies (including BMGF, Centers for Disease Control, USA (CDC), Rotary, UNICEF and WHO). GPEI partners in the region continued to oversee the outbreak response through weekly calls with affected countries and UNICEF Headquarters and UNICEF Supply Division. The office provided technical support for complex matters, coordinated quarterly outbreak assessments, convened the Polio Horn of Africa Technical Advisory group (TAG) meetings, documented and shared best practices and conducted regular reviews of financial resource requirements and needs.

Human, financial and material resources were mobilized and committed to interrupt transmission of these polio viruses. Coordination of the resources and efforts was undertaken centrally by a Horn Africa Coordination Office, based in Nairobi. The office coordinated activities including policy development and support to countries. An independent polio group of monitoring board monitored the use of resources and implementation of programs to eradicate polioviruses in the countries. The WHO Regional Office coordinated the disbursement of resources and technical assistance to the countries as well as outbreak response.

At its 14^th^ meeting in August 2015, The TAG recommended an assessment of the HOA office functions to decide on the future of this mechanism and its use for response to future public health events like the poliovirus outbreak in the HoA. This paper summarizes the findings of the review and provides recommendations, with a view to highlighting their contribution to other public health programmes in the Region. It also highlights best practices in coordination of public health interventions that could be borrowed to benefit programmes.

## Methods

A team of four, (including representation from BMGF, WHO/HQ, WHO/EMRO and UNICEF/HQ), led by an independent consultant conducted a desk review of the coordination mechanism and supplemented this with interviews of technical staff from GPEI Partner agencies based in Nairobi. WHO/AFRO was not represented in the review team to avoid conflict of interest as the Coordinator was a WHO/AFRO employee. The mandates of the HoA coordination team namely close monitoring and implementation of the HoA outbreak response plan, guiding countries to implemet the Technical Advisory Group (TAG) recommendations, outbreak risk assessment recommendations and recommendations from the polio Independent Monitoring Board (IMB) were reviewed and these were used as markers for the assessment of the impact of the coordination office for HoA wild poliovirus outbreak. The assessors also interviewed staff of the country programmes, the HoA offices and community members. The outcomes of the exercise were analyzed qualitatively to conclude and a set of recommendations.

## The Mandate of the HoA Coordination Office

A joint AFRO/EMRO/ESARO meeting took place in Cairo in June 2013. The outcome of the meeting was a plan for outbreak response in two phases. Phase1, which spanned from April to November 2013. During this period the coordination office was expected to interrupt WPV1 transmission in the outbreak zones (initially Somalia and Kenya, but later included Ethiopia); protect populations at risk of the WPV1 outbreak; maximize opportunities for immunization in inaccessible areas of south-east Somalia; and protect populations in other areas that may be at risk due to population movement.

The second phase of the mandate spanned from November 2013 to April 2014. During this phase, the coordination office was expected to interrupt on-going WPV1 transmission in insecure areas of south-central Somalia, by April 2014; interrupt transmission of WPV1 in Somali region of Ethiopia by end of 2013 and sustain protective population immunity in other recently infected areas in Somalia and Kenya; sustain high level of population immunity in areas of high risk; and continue conducting routine vulnerability reduction activities.

## Results

### Epidemiology of the outbreak in the Horn of Africa

**Figure 1:**
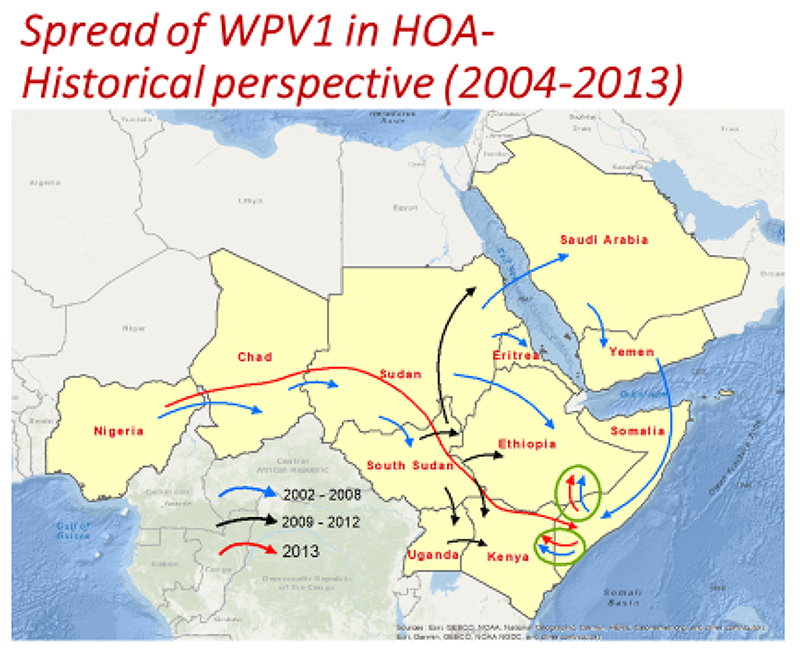
HOA WPV1 Outbreak, 2004-2013

The Horn of Africa (HoA) experienced the most recent outbreak of wild poliovirus type1 (WPV1), which started in April 2013, with detection of a case in Banadir region of Somalia. The virus isolated was confirmed as an importation from Nigeria. The outbreak spread to bordering areas of Kenya and Ethiopia. The last case of the WPV outbreak was detected in Puntland Somalia in August 2014. A total of 223 cases were confirmed (10 in Ethiopia, 14 in Kenya and 199 in Somalia) (Table 1). The countries in the Horn of Africa have experienced previous outbreaks due to importation of WPV1. The most recent outbreaks prior to the current one was from 2004 – 2008 and 2009 - 2012. (Figure 1) The 2013-2014 outbreaks occurred in a complex emergency setting with spread facilitated by inaccessibility, insecurity and massive population movement.

Following effective implementation of plans developed to address the outbreak, the last wild poliovirus was reported on the 11th of August 2014 in Puntland Somalia. Activities to ensure that no transmission was missed continued till the end of 2016 when the coordination office closed.

### Partner Coordination during Outbreak Response

Following detection of WPV1 in Somalia and the declaration of the outbreak in Somalia, the key GPEI partners based in Nairobi held coordination meetings on a weekly basis. These meetings were attended by technical staff from USAID, UNICEF, CDC and WHO. These meetings took place at USAID, UNICEF and WHO Offices. An informal coordination mechanism was therefore established. The key partners (USAID, UNICEF, CDC, BMGF, WHO) each assigned a technical focal point person to participate in the coordination activities. The ten affected countries established a platform for collective decisions to synchronize activities and organize cross border collaboration for campaigns, and to develop the joint WHO/UNICEF HOA Donor Appeal in the fall of 2013.This informal mechanisim was key in coordinating outbreak response activities until a formal coordinating office was established. During Phase 1 of the Outbreak Response, 53 polio campaigns were held in Somalia, Kenya, Ethiopia, Yemen, Djibouti, South Sudan, Sudan, Eritrea and Uganda. Table 2 shows the total number of SIAs conducted during the two phases of the outbreak response.

### HOA Coordination Committee

The first formal Horn of Africa Joint Coordination meeting was held on 4th of February 2014, attended by UNICEF/ESARO, UNHCR, ARC, USAID, CDC and CORE Group and chaired by the WHO HOA Coordinator. (Later, the committee co-opted the Organization for Migration (IOM) and the Regional Mixed Migration Secretariat to ensure inclusion of mobile and migrant populations).

At the first meeting, it was agreed that overall coordination would be the responsibility of the WHO Horn of Africa Coordinator. UNICEF would continue to spearhead the vaccine procurement, cold chain logistics, communication and social mobilization. CDC would focus on development of community-based surveillance. CORE group would focus on cross border coordination issues within the Horn of Africa. The coordination meetings were to be held once every two weeks.

### HOA Coordination Office

The following were the tasks set out for the HOA Coordination office:

1. Coordinate the establishment of a single HOA Polio outbreak response room Nairobi, Kenya; (a) Physical workspace and equipment, (b) Location (b) Logistics support, (d) Personnel for coordination (technical), (e) Support staff, (f) Operations room daily activities.
2. Facilitate close monitoring of implementation of Phase 2 of the HOA Polio Outbreak Response plan through: (a) Ensure at least twice weekly meetings of the Joint Polio Outbreak Response Team (comprising technical officers from BGMF, CDC, USAID, Rotary, UNICEF, WHO), with other partners invited as needed, (b) Ensure members of the Joint Outbreak Response Team prepared and disseminated a standard monitoring template for all HOA countries to prepare and submit weekly updates to the HOA operations room (SIAs, RI, surveillance, resource mob, surge capacity), (c) ensure communication with WHO representatives and other country based officials on importance oftimely submission ofweekly updates from countries to the Outbreak response operations room, (d) Ensure the Joint Outbreak Response team prepared and disseminated a single HOA Situation Report (SitRep) that consolidated information provided in the weekly country specific updates, (e) Provide input into the HOA monthly bulletin, (f) Support strengthening cross-border activities (immunization, surveillance), (g) Monitor implementation of the HOA TAG recommendations
3. Identify and optimize opportunities for continued advocacy at Regional, Sub-regional and Country level.
4. Coordinate donor relations as well as resource mobilization efforts including preparation of regular donor updates as well as donor proposals.
5. Provide monthly updates to the WHO Regional Offices (AFRO and EMRO) as well as UNICEF Regional Offices (ESARO and MENARO).
6. Other tasks as needed, to be taken on by the HOA Coordination Office.

### Establishment of a HOA Polio outbreak response Team, Nairobi, Kenya

Even though the Coordinator had been assigned as Team Leader for the Coordination in the HOA office, and relocated to Nairobi as of February 2014, he kept all his responsibilities as the Polio Surveillance Officer and focal point in the AFRO Inter Country Support Team (IST) for East and Southern sub-region. This created challenges due to overlap between the tasks of HOA (10 countries) and the 14 IST/non HOA countries. The HOA Coordinator worked closely and effectively with the UNICEF ESARO Senior Polio Technical Adviser and ESARO Polio C4D Specialist to ensure alignment and close coordination of all HOA WHO and UNICEF country offices in outbreak response activities. The Coordinator position was set up as temporary. However, it is clear that the functions of coordination would be required until Africa is certified polio free (36 months). At the present time, the office is fully funded only until 31 December 2016. The fact that the Coordinator is in WHO Kenya Office risks giving the perception that the Coordinator responds to the WHO Representative, Kenya.

Technical support staffs (Epidemiologist and Data Manager) were on temporary short-term contracts (3 months contracts). The HOA Office was established and is located within the WHO Kenya office. The advantages included the rapid establishment of the physical office and administrative and logistic support. Office equipment and a vehicle were purchased to strengthen the office. Funding was from BMGF, CDC and WHO. The WHO Kenya Country Office provided logistic and administrative support. The Coordination Office contributes to the budget for running costs of the office. Personnel for coordination (technical support staff) are limited to the coordinator, an epidemiologist and a data manager. The Coordination Office does not have dedicated administrative staff. Until June 2016, when a data manager was recruited for the office, WHO HQ and the WHO Kenya Office provided support for data management. Only one room is allocated to the Coordination Office. The coordinator and the epidemiologist share this office. The data manager works from the EPI WHO Country Team Office. The inadequate office space and lack of dedicated support staff has resulted in the WHO Kenya staff having additional HOA work. Operations room daily activities: During peak activities, The WHO EPI Office was used as the “Operations Room”. This was at the expense of taking away valuable office space from the country office. The office is shared with other staff from the WHO and when meetings are convened for coordination, the WHO conference room or the WRs Boardroom is used.

### Close monitoring of implementation of Phase 2 of the HOA Polio Outbreak Response

Twice weekly meetings of the Joint Polio Outbreak Response Team, comprising technical officers from BGMF, CDC, USAID, Rotary, UNICEF and WHO were scheduled and documented with minutes. The team invited other partners as needed. At the peak of the outbreak period, these meetings were held regularly. However, the frequency of the meetings was reduced when the outbreak was declared closed (August 2015). Communication has been maintained through e-mails, bilateral meetings and phone calls.

Weekly updates from the HoA Countries were submitted regularly to the Operations room. Members of the Joint Outbreak Response Team prepared and disseminated a standard monitoring template for use in all the HoA. This contained information on polio supplementary immunization activities (SIAs), routine immunization (RI), surveillance, resource mobilization, and personnel surge capacity for outbreak response. Since closure of the outbreak, four countries, namely Tanzania, Uganda, Djibouti and Eritrea, stopped submitting the weekly update. Kenya submits the report on monthly basis.

Emphasizing importance of timely submission of weekly reports was done through direct communications from the Coordinator to respective WHO Representatives and other country-based officials. This enforcement was, however, primarily with the outbreak countries. The HoA Situation Report (SITREP): The Joint Outbreak Response team prepared and disseminated a single HoA Situation Report that consolidated information provided in the weekly country specific updates. However, the SITREP was produced only during the outbreak period. It provided input into the HoA monthly bulletin. The bulletin is produced monthly. It summarizes all indicators for acute flaccid paralysis (AFP) surveillance, SIAs and any outbreak response activities that have taken place or are planned in the coming months. Each edition also covered special topics on the outbreak response (for example, results of independent monitoring). The bulletin was produced regularly except on a few occasions when the epidemiologist was on leave. Support for the publication was given by WHO/HQ.

**Figure 2:**
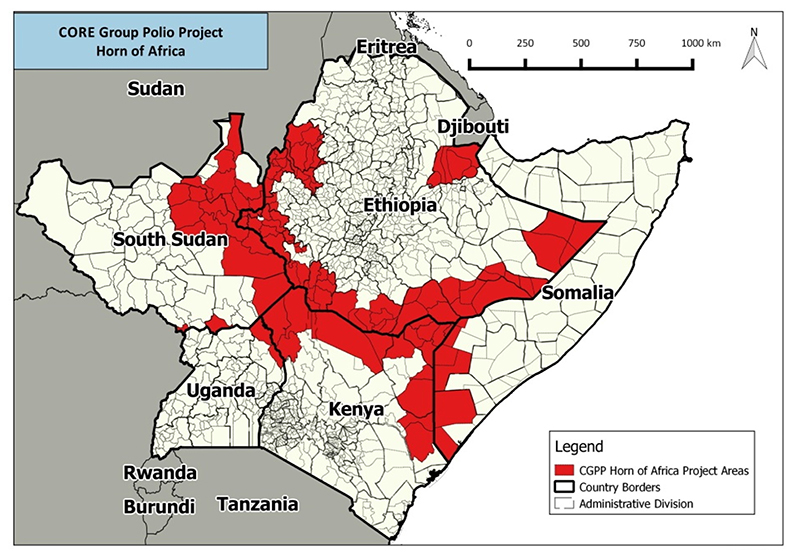
Coordination of Cross-Border Activities (CORE Group Activities)

The HoA Coordination office worked with CORE Group to plan cross-border activities for the outbreak response. Figure 2 shows the areas where the activities have been implemented. The activities promote partnership among border communities and health staff. Examples of achievements include (1) routine EPI outreach logistical support provided to 83 border health facilities focusing on hard to reach areas and nomadic settlements, (2) A total of 6,112 children immunized along Kenya & Somalia border in the Mar/Apr 2016 SIAs, (3) Community Mobilisers recruited and trained on Community Based Disease Surveillance in Somalia (139 CHVs), and Kenya (64 CMs), (4) 5 AFP suspected cases reported since April 2016 (Kenya: 3-Turkana, 2- Kamukunji, and Somalia-1) and (5) In Somalia: 7 villages in El Waaq and Belet Hawa reported inaccessible. CGPP, a local partner of CORE, is working with village leaders for access for Immunization. (Figure 2)

With respect to monitoring implementation of the HoA TAG recommendations, the coordination office collects and collates all information on the status of the implementation of TAG recommendations. Prior to each TAG meeting or Conference call, the Coordination Office works with each country team to prepare full reports of progress on implementation and this information is made available for review by the HoA TAG.

### Identifying and optimizing opportunities for continued advocacy at Regional, sub-regional and country level

Although the office did not perform this function, they utilized the TAG Chairman and members for high level advocacy with partner agencies and countries on specific occasions. An example was the advocacy for use of IPV in N.E. Kenya (Dadaab) during the outbreak.

### Coordinating donor relations as well as resource mobilization efforts including preparation of regular donor updates as well as donor proposals

The HOA office provided regular technical updates on the outbreak and response, through the weekly feedbacks and the monthly bulletin.

### Improving SIA planning and quality

The HOA Office worked with technical partners (CDC) and developed a standard micro planning tool and ensured as much as possible that the dates for SIAs were synchronized and followed up on actual implementation. UNICEF ESARO, in collaboration with IOM, identified and mapped mobile and migrant populations within the HOA and developed communication and behaviour changes strategies to reach these remote populations for vaccination. The office also worked with the countries to implement independent monitoring (IM) and expanding it in Somalia and South Sudan. Lot Quality Assurance (LQAS) was also introduced in selected areas for campaign endprocess monitoring. Figure 2 summarizes the number of SIAs conducted as part of the outbreak response.

### Surveillance reviews and quality

The Office assisted in the coordination of technical support for surveillance reviews. This was done through contacts with partners to participate, identifying technical staff from the key partners (UNICEF, CDC, WHO, BMGF, other WHO Country Offices). This fostered exchange of experiences among the HOA countries.

### Support for laboratory activities

Laboratory support was key during the outbreak. In particular, the National Laboratory in Kenya (KEMRI) supports both Kenya and Somalia, which had the largest number of specimens processed. Although the laboratory was able to fully support the increased workload, the support to laboratory personnel remains inadequate. For ten years, the key technical staff responsible for the testing of specimens has remained on a short-term contract. This issue has been brought to GPEI before but remains unresolved. The current situation poses a risk for performance of the laboratory as the staff could move if he got another more permanent job offer.

### Monitoring Progress

Apart from the regular activities indicated above, closer oversight has been maintained by the Coordination Office for several HOA countries that are a priority or at great risk because of a variety of reasons (including security, lack of infrastructure, very poor performance). For these selected countries, the Office holds regular teleconferences, at least once a month for each. For Somalia, a Dashboard has been set up for monitoring of activities and for Ethiopia; an accountability framework is in place.

### Monthly updates to the WHO Regional Offices (AFRO and EMRO) as well as UNICEF Regional Offices (ESARO and MENARO)

Except for the regular technical updates mentioned above (HOA monthly polio bulletin, weekly situation report) no other monthly updates were provided to the WHO & UNICEF Regional offices. The monthly updates to the Regional Directors of AFRO and EMRO were discontinued after the closure of the outbreaks in August 2015.

### Other tasks taken on by the HOA Coordination Office

Together with WHO HQ, the HOA office supports coordination of all the technical preparations for the HOA TAG. In addition, the coordinator took on the below tasks as IST Polio surveillance officer (a) Implementation of POSE (Polio Outbreak Simulation Exercises) in Sudan, Tanzania, Eritrea and Uganda, (b) Outbreak assessments in South Sudan, Somalia, Kenya and Ethiopia and (c) Surveillance reviews in South Sudan, Ethiopia.

## Discussion, Conclusion and Recommendation

All partners who were involved in the polio coordination mechanism recognize that this process can be used for other priority initiatives in the HoA countries such as strengthening routine EPI in priority districts of the HoA countries as well as overall health systems strengthening. The coordination mechanism allowed partners to identify their areas of work based on their expertise and avoid duplication of efforts at the local level during the outbreak response. Involvement of other partners who worked at lower level including IOM, UNHCR, NGOs, was critical in implementing activities such as SIAs & Surveillance, especially in cross border and hard to reach areas.

The HoA TAG gave credibility to the coordination office and this facilitated the office’s oversight and coordination role with respect to the countries. However, with the closure of the outbreak (August 2015) in the four countries in the HoA a regrettable complacency set in, resulting in lost sense of emergency that negatively impacted the coordination. With the current situation in the African Region (identification of WPV1 in September 2016 which had gone undetected in Nigeria since 2011), the risk to the HoA countries remains and coordination of activities should remain a priority for the GPEI until Africa is certified Polio- Free. The assessment of coordination revealed the many benefits of such coordination system for PEI and other disease control programmes. The coordination process led to synergistic effects that enabled the polio programmes of the respective countries to achieve its objectives, with important spillovers in the coordination of other public health programs including EPI and outbreak management^1^.

Implementing a programme as complex as polio outbreak response in the HoA, where multiple rounds of often countrywide vaccinations should be carried out reliably in difficult even security challenged environments requires a coordinated effort. The Coordination Committee allowed several stakeholders to act collectively and repeatedly to lead the process. Members of the Coordination Committee were conversant with the processes in addressing complex immunization programmes that require joint efforts from different stakeholders, and they have been successful as in the case in polio eradication in the Americas^10,11^. The HoA Coordination Office provided cross agency coordination, which is essential for advocacy, fundraising, strategy development, planning and operations, including monitoring for PEI at country level.

The coordination had important potentially long-lasting public health implications in the region, which need to be followed up even after the polio is eradicated. The interagency mechanisms can be used to coordinate response to other public health issues, especially measles and other immunizable diseases, after polio is eradicated using the same structures^12^. The Coordination structures can be used to address public health hazards including outbreaks and other emergencies like natural disasters.

It is therefore recommended that in view of the current (September 2016) threat from WPV from Nigeria, and the remaining surveillance and immunity gaps in HOA countries, the HOA Coordination Office will need to continue to function at least beyond 2017 to ensure sustainability. The HOA office should continue to be in WHO Kenya Office. However, this should not negatively impact on HOA operations. For effective coordination the HOA Office should be allocated adequate working space, which is not now the case. For effective coordination the minimum staffing level should be maintained (one coordinator, one data manager and one epidemiologist).

Furthermore, in view of the current risks the Joint Polio Outbreak Response team meetings should be reintroduced, and scheduled, at least once every three months.

Countries should continue to submit the weekly updates. The HOA Office should communicate with WHO and UNICEF Representatives of Kenya, Tanzania, Uganda, Eritrea and Djibouti, to reinforce the need for the weekly update. The HOA Office should ensure that the HOA bulletin is produced on a monthly basis, without fail.

Finally, should there be any other future multi-country outbreak, a coordination office of a similar nature should be established to manage the outbreak. This was already implemented in the Lake Chad basin countries where a Lake Chad Coordination Office for Nigeria, Chad, Central Africa Republic, Cameroon and Niger was established with a similar set up. This experience has also been used to establish the Rapid Response Team (RRT) in 2019 based in AFRO to manage the ongoing multi-country outbreak.

## Figures and Tables

**Table 1:** Confirmed Wild Polio Virus cases in the HOA (2001-2015)

Country	Last Indg Case	2001	2002	2003	2004	2005	2006	2007	2008	2009	2010	2011	2012	2013	2014	2015
Uganda	1996	**0**	**0**	**0**	**0**	**0**	**0**	**0**	**0**	**8**	**2**	**0**	**0**	**0**	**0**	**0**
Kenya	1984	**0**	**0**	**0**	**0**	**0**	**2**	**0**	**0**	**19**	**0**	**1**	**0**	**14**	**0**	**0**
Sudan	2001	**0**	**0**	**0**	**120**	**23**	**0**	**1**	**2**	**5**	**0**	**0**	**0**	**0**	**0**	**0**
Ethiopia	2001	**1**	**0**	**0**	**1**	**22**	**17**	**0**	**3**	**0**	**0**	**0**	**0**	**9**	**1**	**0**
Somalia	2002	**7**	**3**	**0**	**0**	**185**	**35**	**8**	**0**	**0**	**0**	**0**	**0**	**194**	**5**	**0**
Yemen	2006	**0**	**0**	**0**	**0**	**478**	**1**	**0**	**0**	**0**	**0**	**0**	**0**	**0**	**0**	**0**
Eritrea	2000	**0**	**0**	**0**	**0**	**1**	**0**	**0**	**0**	**0**	**0**	**0**	**0**	**0**	**0**	**0**
Djibouti	1999	**0**	**0**	**0**	**0**	**0**	**0**	**0**	**0**	**0**	**0**	**0**	**0**	**0**	**0**	**0**
S/Sudan	2002	**1**	**0**	**0**	**8**	**4**	**0**	**0**	**24**	**40**	**0**	**0**	**0**	**0**	**0**	**0**
Tanzania	1996	**0**	**0**	**0**	**0**	**0**	**0**	**0**	**0**	**0**	**0**	**0**	**0**	**0**	**0**	**0**
Total WPVs		**9**	**3**	**0**	**129**	**713**	**55**	**9**	**29**	**72**	**2**	**1**	**0**	**217**	**6**	**0**

**Table 2:** Outbreak response SIAs to WPV and cVDPV, 2013 -Aug 2016

	Somalia	Kenya	Ethiopia	S/Sudan
**Date of onset of last WPV/cVDPV**	**11^th^ Aug 14**	**14^th^ July 13**	**5^th^ Jan 14**	**12 Sept 14**
**Number SIAs in infected area after last WPV**	**11 NIDs** **2SNIDs** **4 HTR** **4SIADs**	**6NIDs** **llSNIDs** **1 Mop up**	**3 NIDs** **l2SNIDs**	**5 NIDs** **2 SNIDs** **3 SIADs** **93 RRM’s**
**Number of SIAs since outbreak**	**21 NIDs** **6 SNIDs** **5 HTR** **10 SIADs** **2 CHDs**	**6 NIDs** **14 SNIDs** **1 Mop up**	**4 NIDs** **18 SNIDs** **1 Mop up** **1 Emergency**	**10 NIDs** **5 SNIDs** **4 SIADs**
**Number of SIAs with tOPV since May 2013**	**1 SIAD** **2 SNIDs** **4NIDs**	**3 SNIDs** **3 NID**	**8 SNIDs** **3 NIDs**	**3 SNIDs** **8 NIDs** **6 SIDAs**
